# An improved medium for in vitro studies of female reproduction and oviposition in *Schistosoma japonicum*

**DOI:** 10.1186/s13071-024-06191-y

**Published:** 2024-03-07

**Authors:** Yanmin You, Xu Chen, Lele Huo, Longlong Chen, Gongwen Chen, Mengjie Gu, Cun Yi, Jipeng Wang, Wei Hu

**Affiliations:** 1 State Key Laboratory of Genetic Engineering, Ministry of Education Key Laboratory of Contemporary Anthropology, School of Life Sciences, Fudan University, Shanghai, China; 2 National Institute of Parasitic Diseases, Chinese Center for Disease Control and Prevention (Chinese Center for Tropical Diseases Research), NHC Key Laboratory of Parasite and Vector Biology, WHO Collaborating Center for Tropical Diseases, National Center for International Research On Tropical Diseases, Shanghai, China; 3 Metabonomics and Systems Biology Laboratory at Shanghai International Centre for Molecular Phenomics, Fudan University, Shanghai, China

**Keywords:** *Schistosoma japonicum*, Culture conditions, m-AB169 (1640), Female sexual development, Egg production

## Abstract

**Background:**

Schistosomiasis is a disease primarily caused by eggs laid by pathogens called schistosomes. Among the schistosome species infecting humans, *Schistosoma japonicum* possesses the largest fecundity; each adult female produces an average of 3500 eggs per day. The lack of proper culture conditions supporting continuous oviposition in vitro has precluded detailed investigation of mechanisms regulating sexual maturation and egg production in *Schistosoma japonicum*.

**Methods:**

We optimized in vitro culture conditions by replacing reagents that are part of the classical ABC169 medium. Fast Blue BB staining and 4′,6-diamidino-2-phenylindole (DAPI) labeling were applied to observe the sexual development status of the females. In vitro RNA interference (RNAi) technology was used to validate the capability of the modified medium. The detection of male β-alanyl-tryptamine (BATT) was conducted using liquid chromatography–mass spectrometry (LC–MS).

**Results:**

Both m-AB169 (1640) and AB169 (1640) media are capable of facilitating the sexual development of paired virgin female *S. japonicum*, as well as sustaining the mature reproductive organs and egg production of adult *S. japonicum* for at least 22 days in vitro. M-AB169 (1640) provided a more stable condition for supporting the sexual maturity of female *S. japonicum*, as evidenced by the consistent initiation of egg production compared with AB169 (1640). Through a comparative analysis of *S. japonicum* and *S. mansoni* in diverse media, we demonstrated that these closely related species display distinct demands for their sexual development and egg production, suggesting a potential influence of nutritional factors on the observed variations in host ranges among different schistosome species. Importantly, we successfully identified the presence of the pheromone β-alanyl-tryptamine (BATT) in *S. japonicum*, previously identified in *S. mansoni*, highlighting its conserved role in schistosome reproductive development. Through the employment of double-stranded RNA (dsRNA) treatment to silence two genes that are involved in either the male (*gli1*, *glioma-associated oncogene homolog 1*) or female (*vf1*, *vitellogenic factor 1*) side in male-induced female reproductive development of *S. mansoni*, we confirmed that the combination of m-AB169 (1640) and RNAi technology has the capacity to facilitate in vitro studies of *S. japonicum*’s reproductive and oviposition processes.

**Conclusions:**

We developed a novel medium, m-AB169 (1640), that not only maintains the mature reproductive organs and continuous oviposition of adult female *Schistosoma japonicum* for up to 22 days but also supports the reproductive development and subsequent egg-laying of virgin females after pairing with male worms. This study provides a valuable in vitro platform for functional studies of the mechanisms underlying the fascinating biology of the female sexual development and egg production of *S. japonicum*, which may accelerate the development of new strategies targeting schistosome egg production.

**Graphical Abstract:**

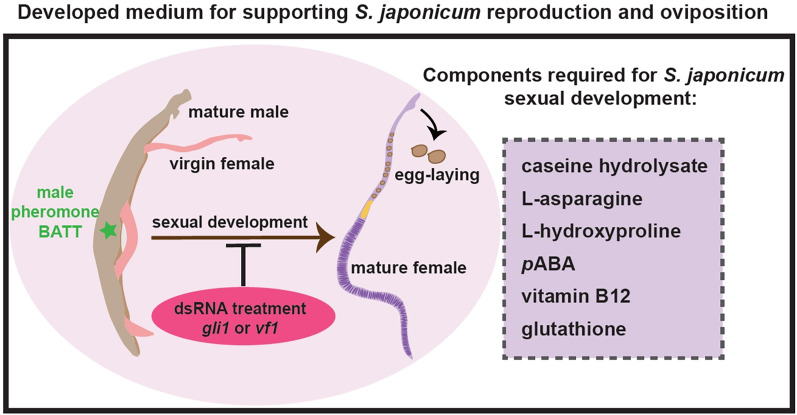

**Supplementary Information:**

The online version contains supplementary material available at 10.1186/s13071-024-06191-y.

## Background

Schistosomes are blood-dwelling parasitic flatworms that infect approximately 250 million people in developing countries [1–3]. The eggs produced by female schistosomes are a critical factor in the spread and pathogenesis of schistosomiasis. Approximately 80% of these eggs become lodged in the host’s organs [7], leading to the formation of granuloma and fibrosis, which cause debilitating symptoms and result in over 250,000 deaths each year [4–9]. The remaining eggs are released into aquatic environments, where they hatch and infect intermediate snail hosts. Therefore, understanding schistosome reproduction and oviposition could lead to new strategies for disease control, as parasites that are unable to produce eggs cause minimal pathology [10, 11].

The use of in vitro cultivation offers controlled conditions that greatly enhance research in the field of life sciences. For schistosomes, several culture media have been developed to sustain these parasites at different stages of their life cycle when they are outside of a host [12–21]. For instance, the Basch medium is optimal for the survival and development of schistosomules in *S. mansoni* [16], while the MEMSE-J medium is commonly used for transferring miracidia into sporocysts [17]. Additionally, Medium 199 (supplemented with 25% FBS) and SBSS (Chernin balanced salt solution) have been proven effective in maintaining sporocysts for up to 14 days [19]. Some of these culture conditions are also widely utilized for functional studies and the evaluation of potential antischistosomal compounds, which greatly contribute to schistosome research [20, 22]. Unfortunately, at present, there is a dearth of any culture medium that can support the growth and development of schistosomes to the same extent as in their natural host. This limitation poses a challenge to the studies of schistosome developmental biology, as their life cycle is complex.

Male-induced female reproduction is one of the most fascinating features of the reproductive biology of schistosomes, and eggs as the outcome of this process are the driving factor of pathology and disease transmission. Previous studies have made significant efforts to establish suitable culture conditions for schistosomes, but only a few have been successful in maintaining normal egg production in adult worms or supporting the sexual development of virgin females in the presence of males [13, 14, 23–26]. Recently, Wang et al. developed a new medium called ABC169 by modifying BM169 (Basch medium 169) with the addition of three nutrients (ascorbic acid, red blood cells, and cholesterol concentrate (ABC)), which has shown promising results in supporting the sexual development and sustained egg production of *S. mansoni* in vitro [13]. This medium was able to mimic the male–female pairing and sexual development that occurs in vivo, leading to the identification of a key signal that is generated by male worms and transferred to female upon pairing, resulting in the subsequent sexual development [12, 13]. However, ABC169 is not suitable for *S. japonicum* sexual development in vitro, which severely limits research on the reproductive development of this particular species. Among the three major schistosome species that cause human schistosomiasis, *S. japonicum* has the highest fecundity. For example, an adult female produces ~ 3500 eggs per day [7], which is almost ten times higher than that of *S. mansoni*. Thus, there is a need for a culture medium that can support the long-term maintenance of the mature sexual organs in adult female *S. japonicum* for continuous egg production or facilitate the reproductive development of virgin females after pairing.

Given that ABC169 has been the most effective way to support the female oviposition of *S. mansoni *in vitro so far, we attempted to develop an equivalent culture condition for *S. japonicum*, using this medium as a starting point. Ascorbic acid in the ABC169 medium, according to Wang et al., is the key factor that guarantees normal egg shape and reproductive development [13, 27], while red blood cells are required to increase egg-laying [13]. Furthermore, adding cholesterol had minimal effects on egg-laying [13], and the cholesterol used in the ABC169 medium is porcine cholesterol concentrate, which is not solely a nutritional source but rather a concentrated form of cholesterol that also contains 8 g/dL proteins and metal ions, as indicated in the product details (https://rmbio.com/products/porcine-cholesterol-concentrate). Therefore, we used AB169 medium (which contains no porcine cholesterol concentrate) as our base medium to create a simpler and more stable medium. In this study, we established two versions of optimized media, m-AB169 (1640) and AB169 (1640), which could not only support the sexual development and egg production of virgin female *S. japonicum* after pairing with males but also maintain the mature status of adult females and continuous egg production. By comparing the performance of *S. japonicum* and *S. mansoni* in different media, we found that the suitable medium for one species may not work for the other, emphasizing that distinct nutritional requirements exist between the two types of schistosomes in vitro. Additionally, we identified the presence of the dipeptide pheromone (BATT) in *S. japonicum* and confirmed its role in inducing female sexual development. Furthermore, we validated the capability of m-AB169 (1640) for in vitro reproductive functional studies using RNAi technology on two genes (*gli1* and *vf1*) [12, 13] associated with female reproductive development. Overall, this work provides valuable in vitro conditions for functional studies of reproduction and oviposition in *S. japonicum*.

## Methods

### Animals and parasites

Female 6-week-old C57BL/6 mice were purchased from Slack Laboratory Animal Co., Ltd. (Shanghai, China). Single-sex infection was administrated by infecting each mouse with cercariae released from an Anhui *Oncomelania* snail challenged with single miracidium, while mixed-sex infection was conducted by infecting each mouse with cercariae released from snails challenged with 10 miracidia. *S. japonicum* (Mainland China strain) or *S. mansoni* (NMRI strain) worms were recovered from the infected mice by perfusion through the hepatic portal vein with cold sterile saline plus heparin (200–350 U/mL) (Solarbio, China). Worms were then rinsed several times in Dulbecco’s modified Eagle medium (DMEM) with 5% fetal bovine serum (FBS) before cultivation.

### Medium formulation

The preparation of BM169 refers to studies of *S. mansoni* [12, 13], using a liquid BME (Basal Medium Eagle) (Gibco) as a substitute for the powder BME in this study. We produced AB169 by adding 200 μM ascorbic acid (Sigma-Aldrich) and 0.2% *V*/*V* bovine washed red bloods cells (10% suspension; Hongquan Bio, Guangzhou, China) into BM169. AB169 (1640) was made by substituting the BME with RPMI-1640 (Gibco) in AB169. M-AB169 (1640) was obtained by replacing half the amount of the lactalbumin hydrolysate (Sigma-Aldrich) in AB169 (1640) with casein hydrolysate (Merck Millipore). M-BM169 (1640) was formulated by replacing BME with RPMI-1640 and incorporating casein hydrolysate in place of half of the lactalbumin hydrolysate present in the original BM169. Detailed compositions of the media are provided in Additional file 7: Table S1.

### In vitro conditions

For regular maintenance, 3–5 worm pairs were cultured at 37 ℃ in 5% CO_2_ in 3 mL medium in a 12-well plate, and the medium was changed every other day. To count egg-laying variation, individual worm pairs were cultured in 2 mL medium in a 24-well plate, and eggs were removed for counting every 24 h. For the BATT treatment experiment, 3–5 mature females or 6–8 virgin females were cultivated in 3 mL medium in a 12-well plate. The medium was replaced daily with renewal of either BATT or dimethyl sulfoxide (DMSO). To count the egg-laying on day (D) 12, eggs in each well were cleaned on D11. To calculate the egg/female/day in cultivation, the total number of eggs produced during the cultivation in each group was counted using ImageJ on captured images of eggs in the dish, and days were calculated since the initiation of egg production. Worms that were separated or disabled during cultivation were removed from the experiment.

### BATT synthesis and detection

BATT has been identified as a pheromone synthesized by males and transferred to females during pairing before triggering downstream reproductive development in *S. mansoni* [12]. To determine whether BATT is conserved within schistosome species on regulating the male-induced female reproduction, we sought assistance from the Chinese Center for Disease Control and Prevention for the chemical synthesis of BATT, as referred to a previous study [12]. Concentrated stock solutions (100 mM) of BATT were prepared in sterile DMSO and stored at –20 ℃. LC–MS was employed for detection of BATT in parasite samples [12, 28]. Thirty male worms after snap-freezing in liquid nitrogen were treated with 200 μL of methanol: water (10:1) and 2 steel balls, followed by tissue homogenization (three cycles, 30 Hz, 30 s) and three freeze–thaw cycles using liquid nitrogen and water. The samples were subjected to frequency-variable ultrasound for 10 min, followed by centrifugation at 18,620*g* for 10 min. Finally, 40 μL of the supernatant was used for BATT detection. BATT detection was performed on a triple quadrupole mass spectrometer operating in multiple reaction monitoring (MRM) employing a negative electrospray ionization (ESI) interface using a QTRAP 6500+ system, with the transitions set at *m*/*z* 232.4 → *m*/*z* 143.7, *m*/*z* 232.4 → *m*/*z* 185.1, and *m*/*z* 232.4 → *m*/*z* 89.1, respectively.

### Parasite staining and imaging

Fast Blue BB staining and DAPI labeling were performed as previously described [12, 13]. Confocal imaging of fluorescent samples was performed on a Nikon A1 laser scanning confocal microscope or Olympus FV3000 laser scanning confocal microscope. Brightfield images were acquired on a Nikon ECLIPSE Ts2 or Zeiss AxioZoom V16 microscope. ImageJ was used to calculate the sizes of ovaries and eggs.

### RNA interference (RNAi)

dsRNA production and RNAi treatment were essentially performed as previously described [12, 13, 29]. Oligo sequences used to generate dsRNA templates, as well as the corresponding dsRNA templates, as outlined in Additional file 8: Table S2. DsRNA of green fluorescent protein (GFP) fragment was used as control for all RNAi experiments. D0 represents the first day of the experiment.

For the *gli1* RNAi experiment, males were treated with 30 μg/mL dsRNA of GFP or *gli1* (on D0/2/4/6 with fresh medium) for 1 week in m-BM169 (1640) and then paired with virgin females from D8 to D28 in m-AB169 (1640) (medium replaced every other day). The eggs produced by the worms were kept in the well when changing the medium. On D28, the number of eggs laid throughout the cultivation period was counted to calculate the egg/female/day (the number of days since the commencement of egg production was considered).

For the *vf1* RNAi experiment, virgin females were treated with 30 μg/mL dsRNA of GFP or *vf1* on D0, 2, 4, and 6 in fresh m-AB169 (1640) supplemented with 100 μΜ BATT and cultured until D19 (medium replaced every other day) when phenotypes were monitored. On D19, the number of eggs laid throughout the cultivation period was counted to calculate the egg/female/day (the number of days since the commencement of egg production was considered).

### Quantitative real-time PCR (qRT-PCR)

All samples were collected on D8 for qRT-PCR detection. All qRT-PCR reactions were performed on a LightCycler^®^ 96 (Roche, Basel, Switzerland) using 2 × SYBR green qRT-PCR master mix (YEASEN, China) according to the manufacturer’s instructions. Each 20 μL qRT-PCR reaction mixture comprised 2 μL of cDNA (1:4), 10 μL 2 × SYBR green master, 0.8 μL (5 μM) of each primer, and 6.4 μL ddH_2_O. The qRT-PCR cycle parameters were as follows: 95 ℃ for 3 min followed by 40 cycles of 95 ℃ for 15 s, 60 ℃ for 30 s; melt curve analysis ranged from 60 ℃ to 95 ℃ to ensure that the specific product was amplified in each reaction. The 2^−ΔΔCt^ method was used to calculate the relative fold change of the differentially expressed transcripts [30]. The endogenous gene *psmd* (*26S proteasome non-ATPase*) served as internal control. All primers for qPCR are listed in Additional file 8: Table S2.

### 5-Ethynyl-2'-deoxyuridine (EdU) staining and hatching assays of eggs

Eggs were labeled with 10 μM EdU for 2 h, and then treated with 4% formaldehyde in PBSTx for 1 h. After washing with PBSTx, 1 mL EdU detection solution was added and incubated for 30 min as in previous report [12]. After incubating the eggs with 1 ug/mL DAPI in PBSTx, we acquired the results by Olympus FV3000 laser scanning confocal microscope.

For the hatching assays of eggs, the eggs were transferred to a new culture dish with Medium 199 and cultured for 9 days. Subsequently, we replaced Medium 199 with dechlorinated water. After 2 h of illumination, we observed miracidia under the microscope.

### Quantification and statistical analysis

GraphPad Prism software was used to process and present the data as mean with standard deviation (SD). Statistical significance was calculated by the unpaired two-tailed parametric *t* test. *P*-values < 0.05 are considered significant (*ns*, not significant; *P < 0.05, **P < 0.01, ***P < 0.001, ****P < 0.0001). Error bars represent SD.

## Results

### M-AB169 (1640) and AB169 (1640) support the sexual development of *S. japonicum* females in vitro

**Fig. 1:**
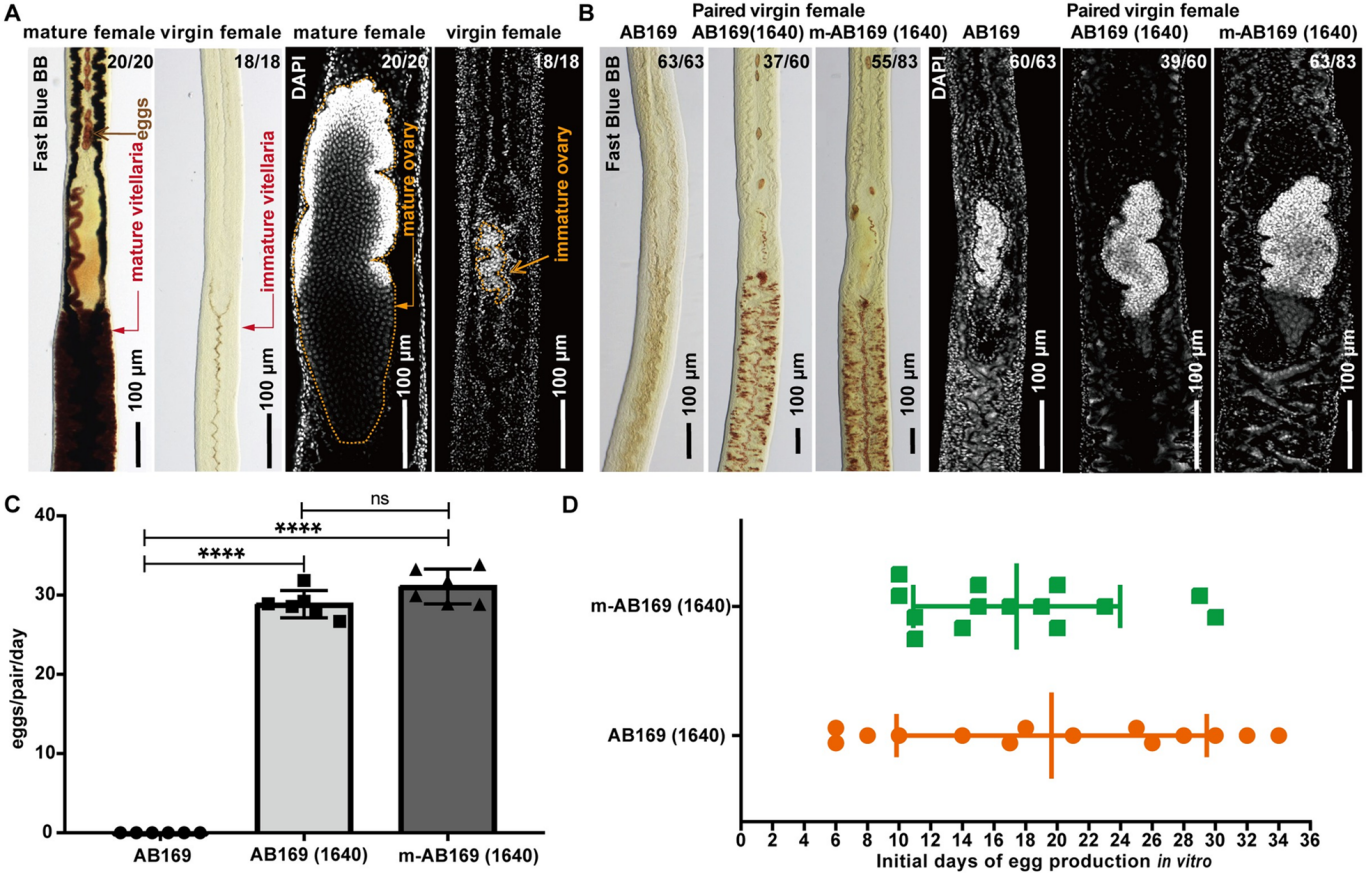
M-AB169 (1640) supports the sexual development of paired virgin *S. japonicum* females. **A** Fast Blue BB staining (red) and DAPI labeling (gray) showing the vitellaria (left) and ovary (right) of the freshly perfused sexually mature females (*n* = 20) and immature virgin females (*n* = 18). Fast Blue BB labels mature vitellocytes. **B** Fast Blue BB staining (red) and DAPI labeling (gray) showing status of vitellaria (left) and ovary (right) in the paired virgin females after culturing in AB169 for 26 days and AB169 (1640) and m-AB169 (1640) for 21 days. Representative images from six experiments with *n* ≥ 60 parasites in each group. **C** Evaluation of egg production of virgin females pairing with males after cultivation in AB169 (D26), AB169 (1640) (D21), and m-AB169 (1640) (D21); Egg production was examined with *n* ≥ 10 worms for each group. The total number of eggs produced during the cultivation in each group was counted for calculation. *n* = 6 biological replicates. *ns*, not significant, *P < 0.05, ***P < 0.001, *t* test. Error bars represent the SD. **D** Days of the initial egg production by worms cultured in AB169 (1640) and m-AB169 (1640). *n* ≥ 3 parasites for each statistic; *n* = 14 biological replicates. Error bars represent the SD

Virgin female schistosomes launch and complete their reproductive development upon pairing with male worms [31–34]. Unlike virgin females, mature females possess fully developed sexual organs including ovary and vitellaria [35, 36], which could be clearly labeled by DAPI or Fast Blue BB (Fig. 1A). Correspondingly, these two staining methods were used to evaluate the status of the schistosome’s reproductive organs [12, 37]. ABC169, a recently developed medium, can support the complete sexual development of immature female *S. mansoni* when paired with male worms in vitro [13]. Here, we used AB169 as a base medium by withdrawing cholesterol from ABC169. To test whether AB169 also works for *S. japonicum*, we co-cultured virgin females with mature males. Despite being physical active and paired, these females failed to develop mature vitellaria and ovaries even after extending the cultivation time to 26 days (Fig. 1B), resulting in no eggs observed (Fig. 1C). These data suggested that *S. mansoni* and *S. japonicum* require different nutrients to support their reproductive development.

To formulate a specific medium suitable for the reproductive development of *S. japonicum*, we opted to modify AB169 medium as it contains nutrients known to be essential for egg production, such as ascorbic acid and red bloods cells [13, 37]. Previous studies have shown that RPMI-1640 supplemented with blood cells can partially support the development of vitellaria in immature *S. japonicum* [37], while casein hydrolysate in 851 medium can enhance egg production by mature *S. japonicum *in vitro [38]. Therefore, we replaced the BME in AB169 with RPMI-1640 to make a new medium as AB169 (1640). Additionally, we made another variation of AB169 (1640) called m-AB169 (1640) by substituting half of the lactalbumin hydrolysate with casein hydrolysate.

After 3 weeks of in vitro cultivation, we found that both AB169 (1640) and m-AB169 (1640) media promoted the sexual maturation and egg production of paired virgin females (Fig. 1B, C). Fast Blue BB staining showed that 61.7% (37/60) females in AB169 (1640) and 66.3% (55/83) females in m-AB169 (1640) displayed well-developed vitellaria (Fig. 1B). DAPI labeling indicated that 65.0% (39/60) females in AB169 (1640) and 75.9% (63/83) females in m-AB169 (1640) exhibited differentiated ovaries (Fig. 1B). Notably, we observed a consistent daily egg production ranging from 26 to 33 eggs in both media (Fig. 1C). As egg production serves as a key indicator of the developed sexual organs, we further evaluated the time it took for the parasite to start laying eggs in these two media. As shown in Fig. 1D, the initial day of egg production was more consolidated in m-AB169 (1640) compared with AB169 (1640) (Fig. 1D), indicating a more stable condition. Moreover, we collected the eggs laid by the developed females for EdU labeling and miracidia hatching assays. As indicated in Additional file 1: Fig. S1, 3.77% of eggs from m-AB169 (1640) and 2.04% of eggs from AB169 (1640) were EdU^+^. Additionally, 1.53% of eggs from m-AB169 (1640) and 1.45% of eggs from AB169 (1640) were capable of hatching miracidia. These findings suggest that m-AB169 (1640) is an optimal medium for supporting the sexual maturity of female *S. japonicum*.

### M-AB169 (1640) maintains the mature reproductive organs and egg production of the adult *S. japonicum*

**Fig. 2:**
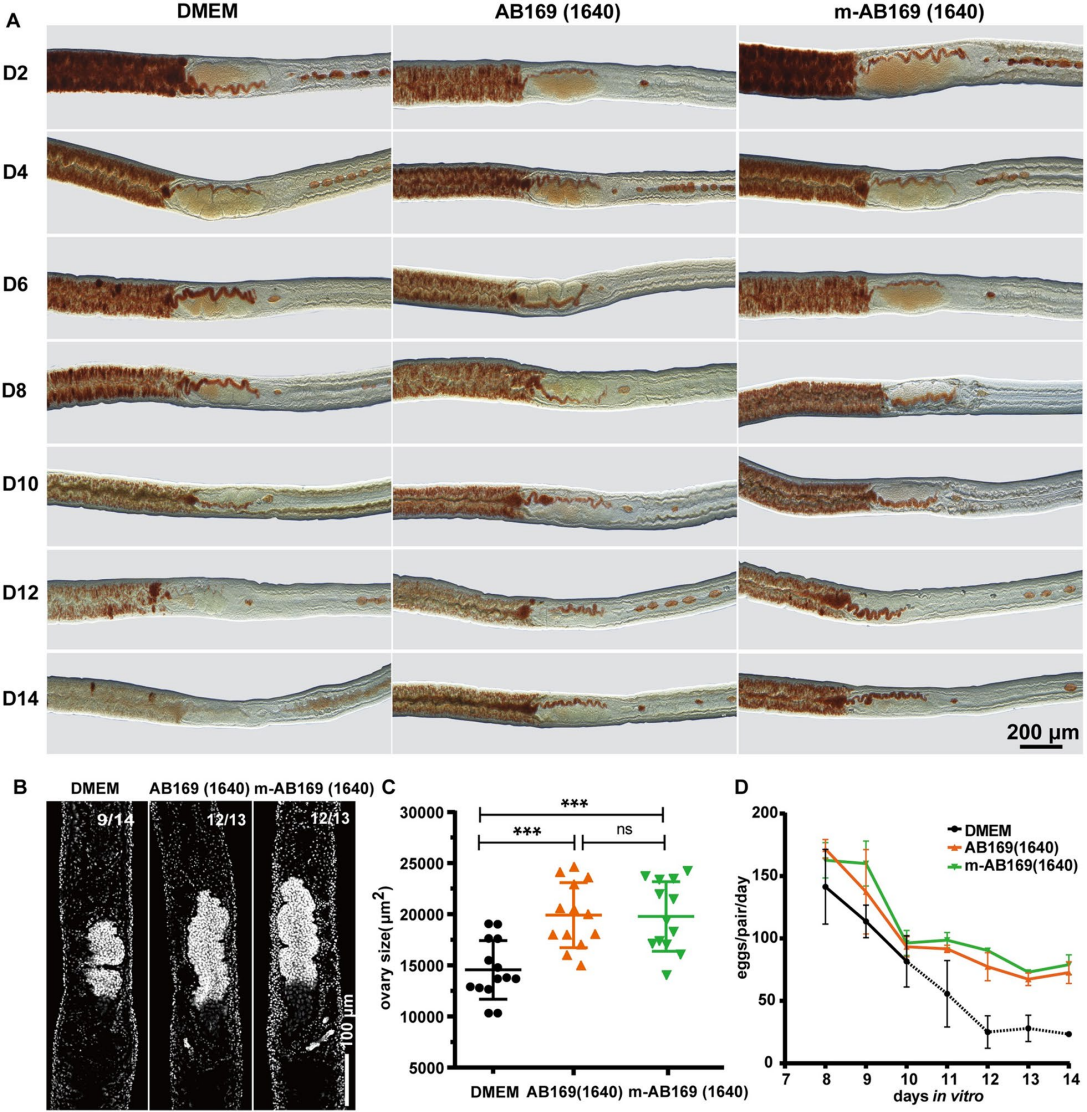
Maintenance of the reproductive organs and egg production of mature *S. japonicum* in different culture media. **A** Fast Blue BB staining (red) showing the changes of vitellaria during cultivation in DMEM, AB169 (1640), and m-AB169 (1640) in 2 weeks. Representative images from three experiments with *n* ≥ 13 parasites for each group. **B** DAPI labeling (gray) showing the ovary of cultured adult female parasites on D14 in DMEM, AB169 (1640), and m-AB169 (1640), respectively; Representative images from *n* ≥ 13 parasites for each group. **C** The sizes (area) of female ovaries measured by ImageJ. *n* ≥ 13 worms for each group. *ns*, not significant, *P < 0.05, **P < 0.01, ***P < 0.001, ****P < 0.0001, *t* test. Error bars represent the SD based on three separate experiments. **D** Rate of daily egg production per worm pair maintained in medium DMEM, AB169 (1640), and m-AB169 (1640), respectively. Dotted line indicates parasites cultured in DMEM begin laying morphologically abnormal eggs from D10. *n* = 24 worm pairs in each group in three experiments, Error bars represent 95% confidence intervals

In many studies, freshly harvested adult worms from hosts were used to perform functional studies or drug efficiency evaluation on the reproduction and oviposition of schistosomes under controlled conditions [12, 13, 20, 22]. However, commonly used media such as DMEM medium (supplemented with 10% FBS) was unable to maintain the mature status of female sexual organs. As shown in Fig. 2A, the vitellaria of adult female *S. japonicum* degenerated faster in vitro and most of the mature vitellarium were lost within 2 weeks of cultivation as indicated by Fast Blue BB staining. Therefore, the availability of a medium capable of maintaining mature reproductive organs and facilitating long-term egg production in vitro becomes essential. Here, we monitored the changes in sexual organs and egg-laying activity of paired adult female *S. japonicum* in DMEM, AB169 (1640), and m-AB169 (1640), since AB169 medium cannot support the sexual development of female *S. japonicum*. Apparently, DMEM medium failed to maintain the mature status of vitellaria and ovaries (Fig. 2A–C), resulting in loss of their ability to lay eggs (Fig. 2D). Conversely, the other two optimized media maintained the mature vitellaria even after a 22-day in vitro cultivation, as indicated by a strong signal using Fast Blue BB staining (Fig. 2A; Additional file 2: Fig. S2). Taken together, both AB169 (1640) and m-AB169 (1640) exhibited similar and robust effects on sustaining the mature sexual organs of adult female *S. japonicum*. Given that m-AB169 (1640) slightly outperformed the AB169 (1640) in supporting the sexual development and egg production of paired virgin females, we ultimately employed m-AB169 (1640) for subsequent experiments.

### Mature *S. japonicum* and *S. mansoni* require distinct culture conditions

**Fig. 3:**
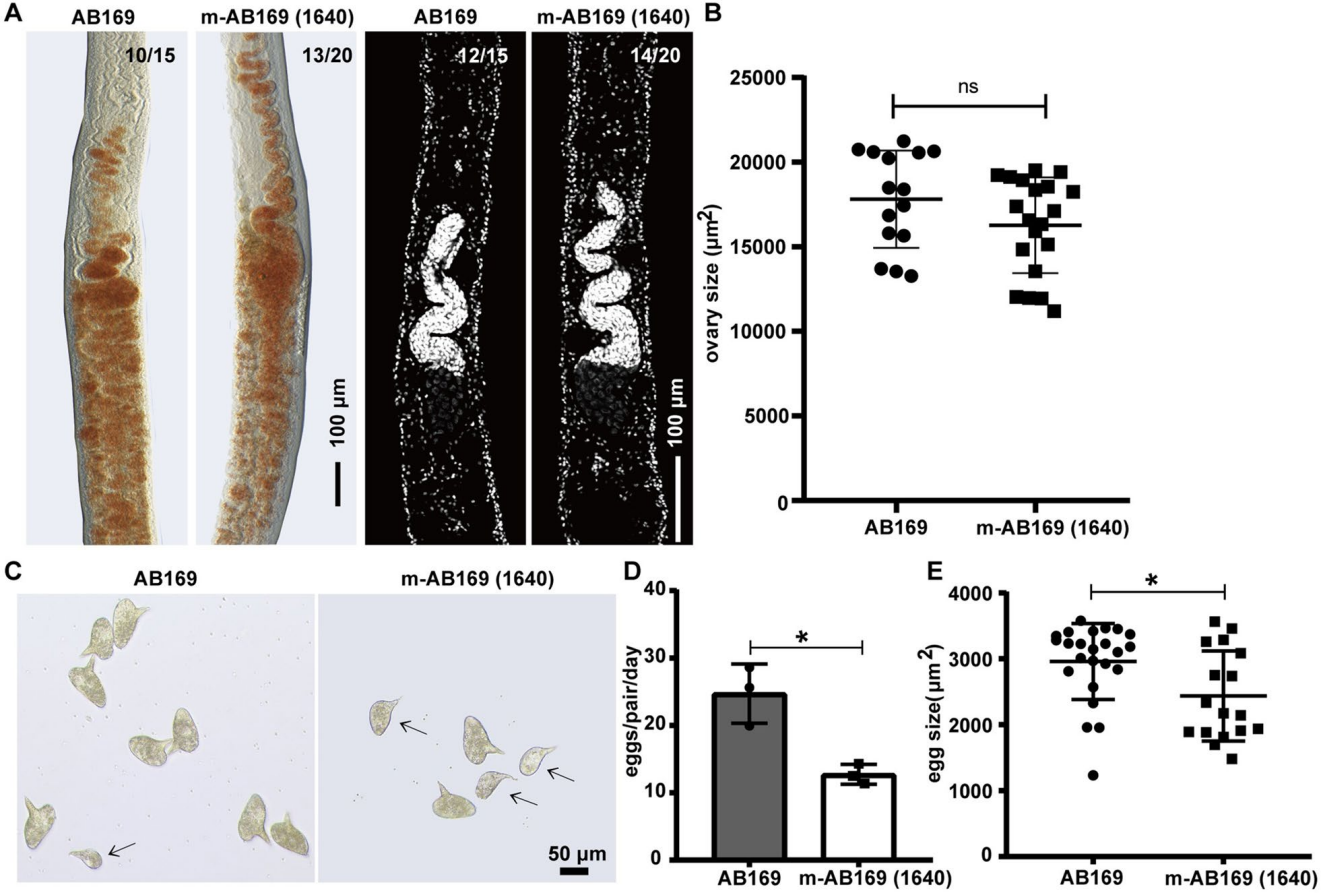
Ineffectiveness of m-AB169 (1640) in maintaining the oviposition of *S. mansoni* compared with AB169. **A** Fast Blue BB staining (red) and DAPI labeling (gray) showing the vitellaria (left) and ovary (right) of adult female *S. mansoni* cultured in AB169 and m-AB169 (1640) on D12. Representative images from three experiments with *n* ≥ 15 parasites. **B** The sizes (area) of female ovaries. *n* ≥ 15 worms for each group. **C** Eggs laid by paired adult females cultured in AB169 and m-AB169 (1640) on D12. The arrows indicate the presence of malformed eggs. Representative of three experiments. **D** Eggs laid per day per paired parasite cultured in AB169 and m-AB169 (1640) on D12. *n* ≥ 15 females for each group. **E** Sizes of eggs laid by parasites cultured in AB169 (*n* = 24) or m-AB169 (1640) (*n* = 17) on D12. *ns*, not significant, *P < 0.05, *t* test. Error bars represent the SD

When comparing m-AB169 (1640) and AB169, it was observed that m-AB169 (1640) contains more nutrients than AB169. Further experiments were done to determine whether m-AB169 (1640) could be used to cultivate *S. mansoni* as well. Consistent with the report by Wang et al., AB169 successfully maintained the mature vitellaria and ovary of adult *S. mansoni* after 12 days of in vitro cultivation (Fig. 3A). However, m-AB169 (1640) was unable to prevent the vitelline from degeneration, despite no differences in ovary size between the two media (Fig. 3B). Eggs laid by the parasite on day 12 in AB169 exhibited typical features, including well-defined lateral spines (Fig. 3C). In contrast, small eggs and reduced egg production were observed in the m-AB169 (1640) group on day 12 (Fig. 3C–E). These findings indicated that m-AB169 (1640) was able to support maintaining the differentiation status of the sexual organs of *S. japonicum* females but not of *S. mansoni* females. Our previous data (Figs. 1B, C and 2) also suggest that the medium suitable for one schistosome species has little to no effect on the other species, highlighting the different nutritional requirements for reproduction and oviposition in *S. japonicum* and *S. mansoni*.

### BATT is conserved in schistosome species on regulating male-induced female reproduction

**Fig. 4:**
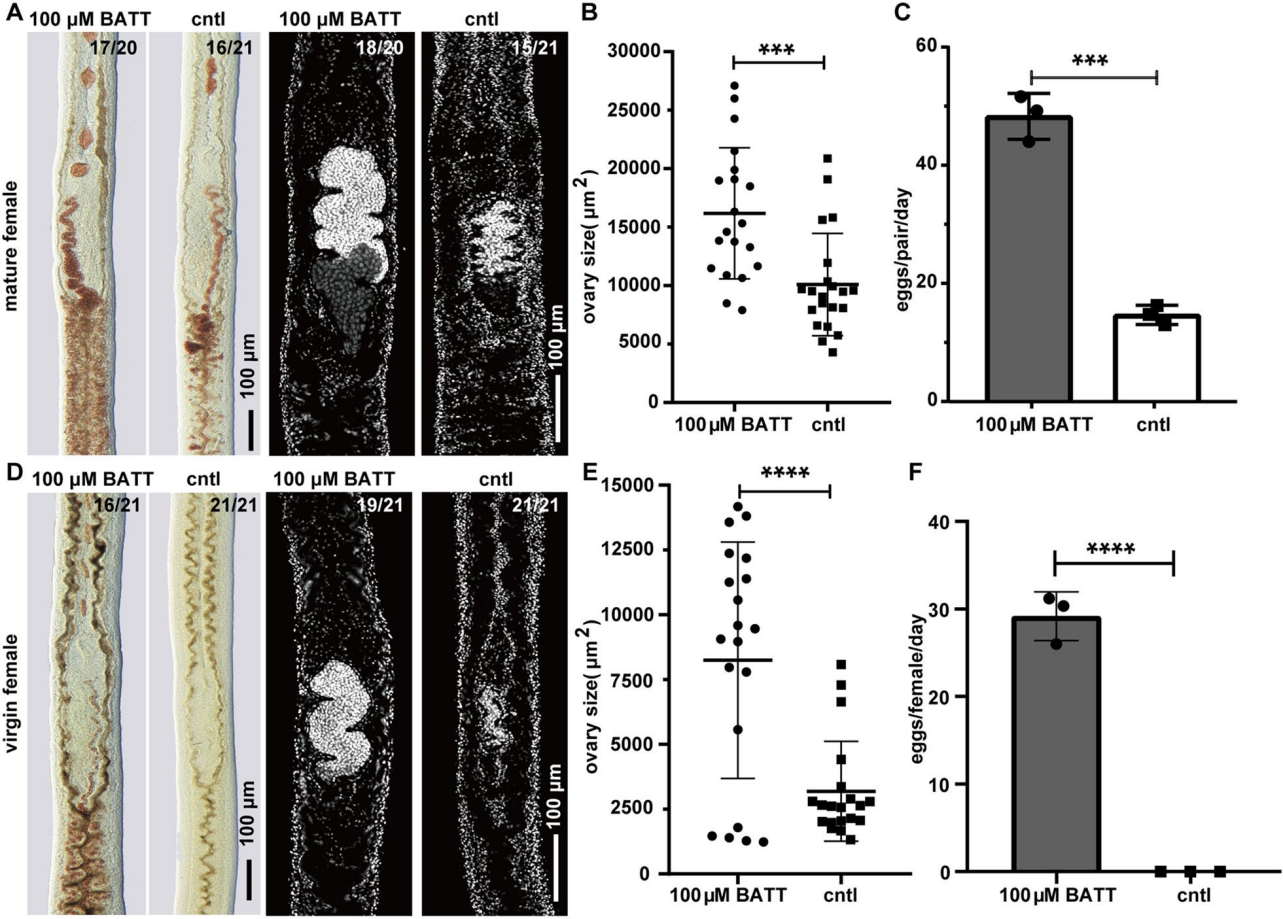
BATT induces the reproductive development and egg production of *S. japonicum*. **A** Fast Blue BB staining (red) and DAPI labeling (gray) showing the vitellaria (left) and ovary (right) of unpaired adult *S. japonicum* females cultured in m-AB169 (1640) supplemented with BATT or DMSO on D12. Representative images from three experiments with *n* ≥ 20 parasites for each group. **B** The sizes (area) of the ovaries in adult female cultured in m-AB169 (1640) with BATT or DMSO on D12. *n* ≥ 20 females for each group. **C** Eggs laid per day per female parasite on D12 of the BATT treatment group and control group. *n* ≥ 20 females for each group. To count the eggs laid on D12, eggs in the wells were cleared on D11. **D** Fast Blue BB staining (red) and DAPI labeling (gray) showing the vitellaria (left) and ovary (right) of unpaired virgin *S. japonicum* females cultured in m-AB169 (1640) supplemented with BATT or DMSO on D26. Representative images from three experiments with *n* = 21 parasites for each group. **E** The sizes (area) of the ovaries in virgin females cultured in m-AB169 (1640) with BATT or DMSO on D26. *n* = 21 females for each group. **F** Eggs laid per day per female parasite in 26 days of the BATT treatment group and control group. *n* = 21 females for each group. The total number of eggs produced in 26 days was counted for calculation. **P < 0.01, ***P < 0.001; ****P < 0.0001, *t* test; Error bars represent the SD based on three separate experiments

Researchers have proposed that male schistosomes induce female sexual development by providing a certain stimulus upon pairing, such as chemicals [12, 39–41]. Chen and Wang et al. recently identified BATT as a key dipeptide synthesized by males and transferred to females before triggering downstream reproductive development in *S. mansoni* [12]. However, it remains uncertain whether BATT is present in other schistosome species. Here, we identified BATT in *S. japonicum* using LC–MS analysis (Additional file 3: Fig. S3). To further test whether BATT could replace male worms to stimulate female sexual development and egg-laying in *S. japonicum* as reported in *S. mansoni*, we cultured the adult female *S. japonicum* from mixed-sex infection or virgin female *S. japonicum* from single-sex infection in our newly developed medium m-AB169 (1640) supplemented with 100 μM BATT. After 12 days of cultivation, BATT effectively prevented the degeneration of the vitellaria and ovary in the adult females (Fig. 4A, B). In the control group without BATT, only a few eggs were produced, while the parasites treated with BATT laid significantly more eggs (Fig. 4C). Moreover, BATT effectively induced the reproductive development of virgin female in vitro. After 26 days of cultivation, the majority of females in the BATT group developed ovaries (19/21) and vitellaria (16/21), and eggs were observed (Fig. 4D–F). In contrast, the control group showed no signs of development and no eggs were observed (Fig. 4D–F). In addition, we observed 3.54% of eggs from mature females (D12), and 2.13% of eggs from virgin females (D26) were able to develop as proliferating cells detected through EdU labeling, though these eggs failed to hatch miracidia (Additional file 4: Fig. S4). These findings not only demonstrate that BATT plays a conserved role in stimulating reproductive development in both *S. japonicum* and *S. mansoni*, but also suggest the potential for using *S. japonicum* as a model to explore schistosome reproduction in our newly developed medium called m-AB169 (1640).

### M-AB169 (1640) serves as a platform for functional studies of schistosome reproduction

To examine the potential of m-AB169 (1640) for schistosome reproduction studies using *S. japonicum*, we chose *gli1* (*Sjc-0005045*) and *vf1* (*Sjc-0000108*), which are highly associated with this process [12, 13], and performed RNAi.

**Fig. 5:**
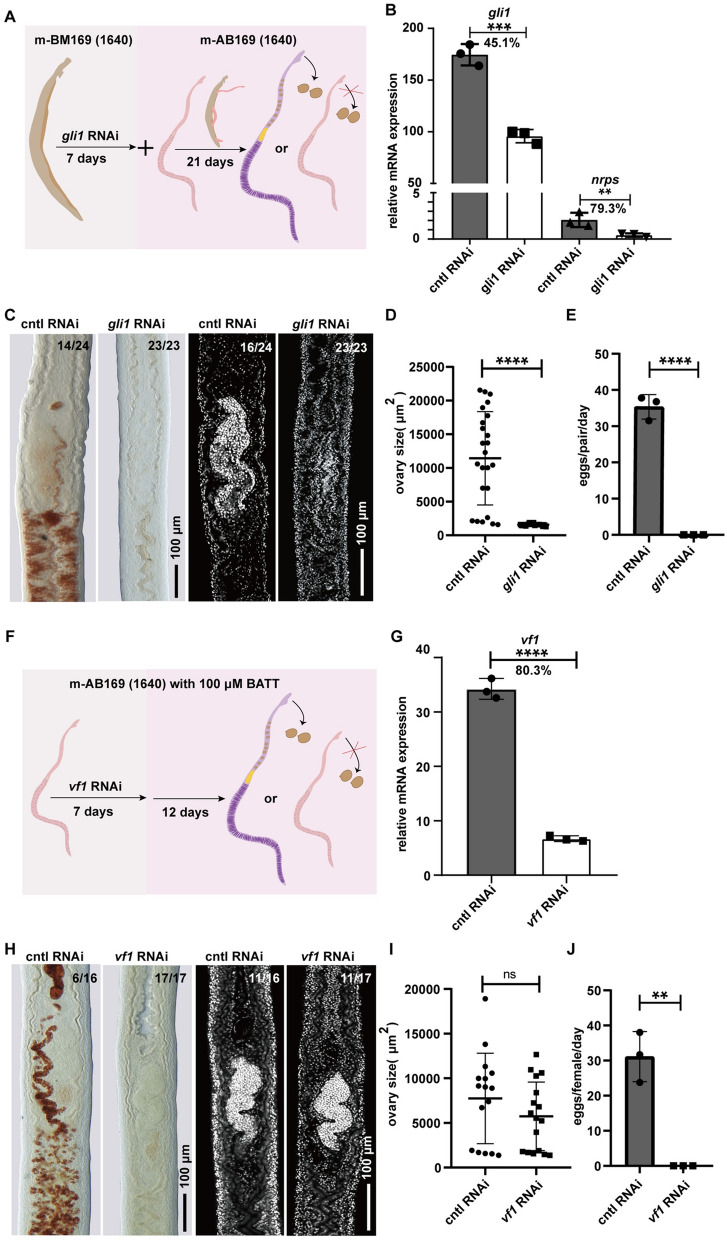
m-AB169 (1640) serving as an RNAi platform for functional studies on the reproduction of *S. japonicum*. **A** Schematic of male *gli1* RNAi experiments. **B** qPCR showing expression levels of *gli1* and *gli1*-dependent *nrps* in the *gli1* RNAi group and cntl RNAi group. **C** Fast Blue BB (red) and DAPI labeling (gray) showing the vitellaria (left) and ovary (right) from female parasites on D28 after pairing with *gli1* RNAi or control RNAi male worms. *n* ≥ 3 experiments with *n* ≥ 23 worms for each group. **D** The sizes (area) of the female ovaries from *gli1* RNAi group and cntl RNAi group. *n* ≥ 23 females for each group. **E** Eggs laid per day per female parasite after pairing with the *gli1* RNAi or cntl RNAi males. *n* ≥ 23 females for each group; The total number of eggs produced in 26 days was counted for calculation. **F** Schematic of female *vf1* RNAi in m-AB169 (1640) supplemented with 100 μΜ BATT. **G** qPCR showing expression levels of *vf1* in *vf1* RNAi group and control RNAi group. **H** Fast Blue BB (red) and DAPI labeling (gray) showing the vitellaria (left) and ovary (right) from the *vf1* RNAi and cntl RNAi female parasites on D19. *n* ≥ 3 experiments with *n* ≥ 16 worms for each group. **I** The sizes (area) of ovaries from the *vf1* RNAi and control RNAi female parasites. *n* ≥ 16 females for each group. **J** Eggs laid per day per female parasite of *vf1* RNAi and control RNAi female parasites. *n* ≥ 16 females for each group. The total number of eggs produced in 19 days was counted for calculation. *ns* not significant, **P < 0.01; ***P < 0.001; ****P < 0.0001,* t* test; Error bars represent the SD based on three separate experiments

In *S. mansoni*, *gli1* (*Smp_266960*) is required for the expression of *nrps* (*Smp_158480*), which encodes an enzyme SmNRPS (nonribosomal peptide synthetase) that produces BATT in male worms to stimulate female reproductive development [12]. We silenced its expression in *S. japonicum* by treating the male worms with dsRNA for a week, followed by pairing with virgin females for 3 weeks in m-AB169 (1640) (Fig. 5A). The *gli1*-knockdown male worms showed a significant reduction in *gli1* expression by 45.1%, and a subsequent 79.3% reduction in *nrps* (*Sjc-0000814*) expression since it is *gli1* dependent (Fig. 5B) [12]. Additionally, the *gli1*-knockdown male worms were unable to induce the virgin female to reach sexual maturation or subsequent egg production (Fig. 5C–E).

In addition to the gene that functions in males, there is a transcription factor called *vf1* (*vitellogenic factor 1*, *Smp_248100*) that is specifically expressed in the vitellaria and is essential for the differentiation of the S1 vitelline cell in *S. mansoni* [13]. In this study, we treated virgin female *S. japonicum* with *vf1* dsRNA or control dsRNA for 1 week in m-AB169 (1640) supplemented with 100 μM BATT, and then cultured them for another 12 days before assessing their reproductive development and oviposition (Fig. 5F). As a result of this treatment, the expression of *vf1* was significantly reduced by 80.3% (Fig. 5G). Staining of the reproductive organs showed that silencing *vf1* hindered the differentiation of the vitellaria of virgin female *S. japonicum* induced by BATT, yet it did not affect the maturation of the ovary (Fig. 5H and I). owing to the absence of the mature vitellaria, which provides the materials necessary for egg formation, no eggs were observed in the *vf1* RNAi group (Fig. 5J). These findings highlight the reliable use of m-AB169 (1640) as an in vitro platform for studying the impact of female genes on reproductive development.

In conclusion, our newly developed medium m-AB169 (1640) could serve as a valuable platform for functional studies of the mechanisms underlying the biology of the male-induced female sexual development and egg production of *S. japonicum *in vitro.

## Discussion

The long-standing objective of maintaining the complete life cycle of schistosomes outside their hosts in a cultured environment has yet to be achieved. The sexual reproduction process of this parasite is particularly captivating, but there is currently a lack of suitable media to support the in vitro female sexual development and egg-laying of *S. japonicum*. In this study, we developed two optimized media for *S. japonicum*, namely m-AB169 (1640) and AB169 (1640). Out of these two media, m-AB169 (1640) exhibited remarkable efficacy in supporting the sexual development of virgin females, as well as maintaining the mature state of adult females and egg production in *S. japonicum*.

Interestingly, we found that AB169 did not have the same effect on the reproductive development of *S. japonicum*. Additionally, m-AB169 (1640), which did support the reproductive development of *S. japonicum*, did not show the same effect on *S. mansoni*. This suggests that these two species of schistosomes may have different nutritional requirements for their reproductive development and egg production. It is worth noting that *S. japonicum* and *S. mansoni* have significant biological differences. For example, adult female *S. japonicum* are larger in size compared with *S. mansoni*, with nearly twice the length of the worms and three times the ovary size (Additional file 5: Fig. S5A–D). Moreover, the daily egg production of *S. japonicum* is ten times that of *S. mansoni*, while the eggs of *S. mansoni* are larger in comparison (Additional file 5: Fig S5E, F) [7, 42]. These distinct biological features, particularly in reproduction and oviposition, may be related to the different nutritional requirements and explain the different performance in different media. On the other side, it is important to note that both *S. japonicum* and *S. mansoni* are zoonotic parasites and exhibit varying natural definitive host species [43]. Our findings on the differential nutritional needs during reproductive system development may provide a partial explanation for the distinct host ranges observed in these closely related schistosomes.

In contrast to AB169, both m-AB169 (1640) and AB169 (1640) were able to promote the sexual development and maintain the mature sexual organs of *S. japonicum*. The differences in composition (Additional file 7: Table S1) between these media suggest that there may be certain nutritional requirements necessary for the reproduction of female *S. japonicum*. As shown in Additional file 9: Table S3, there are nutrients that were either with a higher concentration or unique in the two optimized media than AB169, including some amino acids, i-inositol, choline chloride, casein hydrolysate, and B-complex vitamins. Among the 11 compounds, there is at least a twofold difference in concentration. For example, the concentration of inositol in m-AB169 (1640) and AB169 (1640) was almost 17.5-fold higher than that of AB169 (Additional file 9: Table S3). Notably, inositol-derived metabolites have been demonstrated to be crucial for growth and development of *Caenorhabditis elegans* [44].

Six supplements, including l-asparagine, l-hydroxyproline, *para*-aminobenzoic acid (*p*ABA), vitamin B12, glutathione, and casein hydrolysate, were absent from AB169 (Additional file 9: Table S3). Previous studies have shown that l-hydroxyproline and glutathione are associated with nematode growth and reproduction, mobility, and neurotransmission [45]. As a component essential for the synthesis of folate, *p*ABA is required for the development of parasites, such as *Brugia malayi* larvae and malaria [46, 47]. Additionally, delayed growth and infertility of *C.elegans* is observed when there is a deficiency in vitamin B12 [48–50]. The 851 medium was optimized by replacing lactalbumin hydrolysate with casein hydrolysate, which resulted in an increase in egg production in adult *S. japonicum* [21]. Moreover, this modification extended the duration of the peak egg-laying period and reduced malformed eggs [21]. Our study revealed that casein hydrolysate stabilized the oviposition timing in *S. japonicum* (Fig. 1D). Nevertheless, the relationship between each nutrient and the female growth or fecundity of schistosomes needs to be further investigated. Around 70% of the cultured virgin female *S. japonicum* worms can develop sexually, but their egg production is much lower than that of in vivo females (Fig. 1B, C). This highly suggests that some essential factors for schistosome reproduction and oviposition are missing from the current medium. Thus, understanding the nutritional requirements of schistosomes would accelerate the establishment of in vitro conditions with full capacity in supporting parasite growth and development outside the host.

Our research has some limitations that should be acknowledged. Firstly, we employed *gfp* dsRNA as the control. Previous research on *S. mansoni* has indicated that *gfp* can cause changes in the expression of related genes [51]. Therefore, it is important to carefully choose appropriate controls that have been used in *S. japonicum*. Secondly, the concentration of dsRNA used in our functional studies was 30 µg/mL. However, other studies have reported higher concentrations, such as 50 µg/mL or even higher [52]. Therefore, it is recommended to adjust the concentration according to the knockdown efficiency of the target gene. In regards to BATT, we successfully determined the molecular weight of BATT from *S. japonicum*, which matched that of *S. mansoni* [12]. We are uncertain whether there are any differences in the configuration of BATT between the two species. Considering that BATT is a simple dipeptide, we speculate that they have the same configurations. Since *S. japonicum* is larger than *S. mansoni*, we used a concentration of 100 µM BATT, which is twice the concentration used for treating *S. mansoni*, to enhance the reproductive development of female *S. japonicum*. However, when the concentration was increased to 150–200 µM, the reproductive state of the worms did not appear to be favorable (Additional file 6: Fig. S6). We suspect that this may be attributed to the high concentration of the solvent DMSO used.

## Conclusions

Taken together, this study has established a new medium, m-AB169 (1640), which effectively facilitates the reproductive development and continuous egg-laying of female *S. japonicum*. These findings highlight the particular culture requirements crucial for the sexual development of *S. japonicum*. Importantly, by utilizing RNAi in combination with m-AB169 (1640), we have been able to investigate the reproduction and oviposition in *S. japonicum *in vitro. Therefore, our study offers an important and useful in vitro platform for exploring the fascinating biology of female sexual development and egg production in *S. japonicum*.

### Supplementary Information

**Additional file 1: Figure S1. **EdU staining and the hatching ability of eggs produced by paired virgin females cultured in m-AB169 (1640) and AB169 (1640). **A** EdU staining; **B** EdU^+^ and hatching ratios of the eggs. **C** Miracidia in the egg. **D** Miracidia hatched from the eggs.

**Additional file 2: Figure S2. **Differentiated state of the females at D22.

**Additional file 3: Figure S3. **BATT detected in male *S. japonicum *worms by LC-MS. **A** LC–MS detection of *m*/*z* at 143.7, 185.1 and 89.1 showing that the retention time (5.19) of the peaks of BATT standards (*m*/*z* = 232.4). **B** LC-MS detection of *m*/*z* at 143.7, 185.1, and 89.1 showing that the retention time (5.18) of the peaks from the male *S. japonicum *extracts. The retention time of the three peaks corresponds to that of the BATT standards. *y* axis represents intensity, cps, and *x* axis represents retention time from 0 to 10.0 min. Time is shown for all peaks. *m*/*z*, mass-to-charge ratio. Related to Fig. 4.

**Additional file 4: Figure S4. **EdU staining and the hatching ability of eggs produced by females cultured in m-AB169 (1640) supplemented with 100 μM BATT. **A** EdU staining of eggs produced by mature females on D12; **B** EdU staining of eggs produced by virgin females on D26. **C** EdU^+^ and hatching ratios of the eggs.

**Additional file 5: Figure S5.** Differences in worm length, ovary size, and egg size between* S. mansoni *and *S. japonicum*. **A** Brightfield images showing the adult* S. mansoni *(42 dpi, *n* = 20) and *S. japonicum *(34 dpi, *n* = 21) females stained by Fast Blue BB. **B** Length of adult *S. japonicum* (34 dpi, *n* = 21) and* S. mansoni *(42 dpi, *n* = 20) females. **C** DAPI labeling (gray) showing the ovaries of the adult* S. mansoni *(42 dpi, *n* = 20) and *S. japonicum *(34 dpi, *n* = 21) females. **D** The sizes (area) of ovaries in adult *S. japonicum *and* S. mansoni*. *n* ≥ 20 females for each group. **E** Freshly laid eggs (DAPI-labeled, blue) by adult *S. japonicum *(34 dpi, *n* = 21) and* S. mansoni *(42 dpi, *n* = 20) on D1 after harvested from the mice. **F** The sizes (area) of eggs laid by adult *S. japonicum *and* S. mansoni*. *n* = 13 eggs for each group. ****P < 0.0001, *t* test. Error bars represent the SD.

**Additional file 6: Figure S6. **Mature females cultured (within BATT or DMSO) for 12 days in vitro after separated with males.

**Additional file 7: Table S1. **Detailed compositions of the media in this study.

**Additional file 8: Table S2. **Primers and sequences related to experimental procedures.

**Additional file 9: Table S3. **The concentration (mg/L) of representative components that are different in AB169 (1640), m-AB169 (1640), and AB169.

## Data Availability

The data that support the findings of this study are available in the paper and its supplementary materials (Additional files 1, 2, 3, 4, 5, 6, 7, 8 and 9).

## Abbreviations

ABC: Ascorbic acid, Red blood cells, and Cholesterol concentrate
BATT: β-alanyl-tryptamine
BM169: Basch medium 169
LC–MS: Liquid chromatography–mass spectrometer
dpi: Days post infection
FBS: Fetal bovine serum
*p*ABA: *para*-aminobenzoic acid
RNAi: RNA interference
qRT-PCR: Quantitative real-time polymerase chain reaction
GFP: Green fluorescent protein
DMSO: Dimethyl sulfoxide
SBSS: Hernin balanced salt solution
EdU: 5-Ethynyl-2′-deoxyuridine
NRPS: Nonribosomal peptide synthetase
*gli1*: *glioma-associated oncogene homolog 1*
*vf1*: *vitellogenic factor 1*
DAPI: 4′,6-diamidino-2-phenylindole
dsRNA: Double-stranded RNA
BME: Basal Medium Eagle
DMEM: Dulbecco’s modified Eagle medium
MRM: Multiple reaction monitoring
ESI: Negative electrospray ionization
