# Supplementary figures and images for: An improved medium for in vitro studies of female reproduction and oviposition in Schistosoma japonicum
Source: Parasit Vectors. 2024 Mar 7;17:116. doi: 10.1186/s13071-024-06191-y (PMC10918852; doi:10.1186/s13071-024-06191-y)

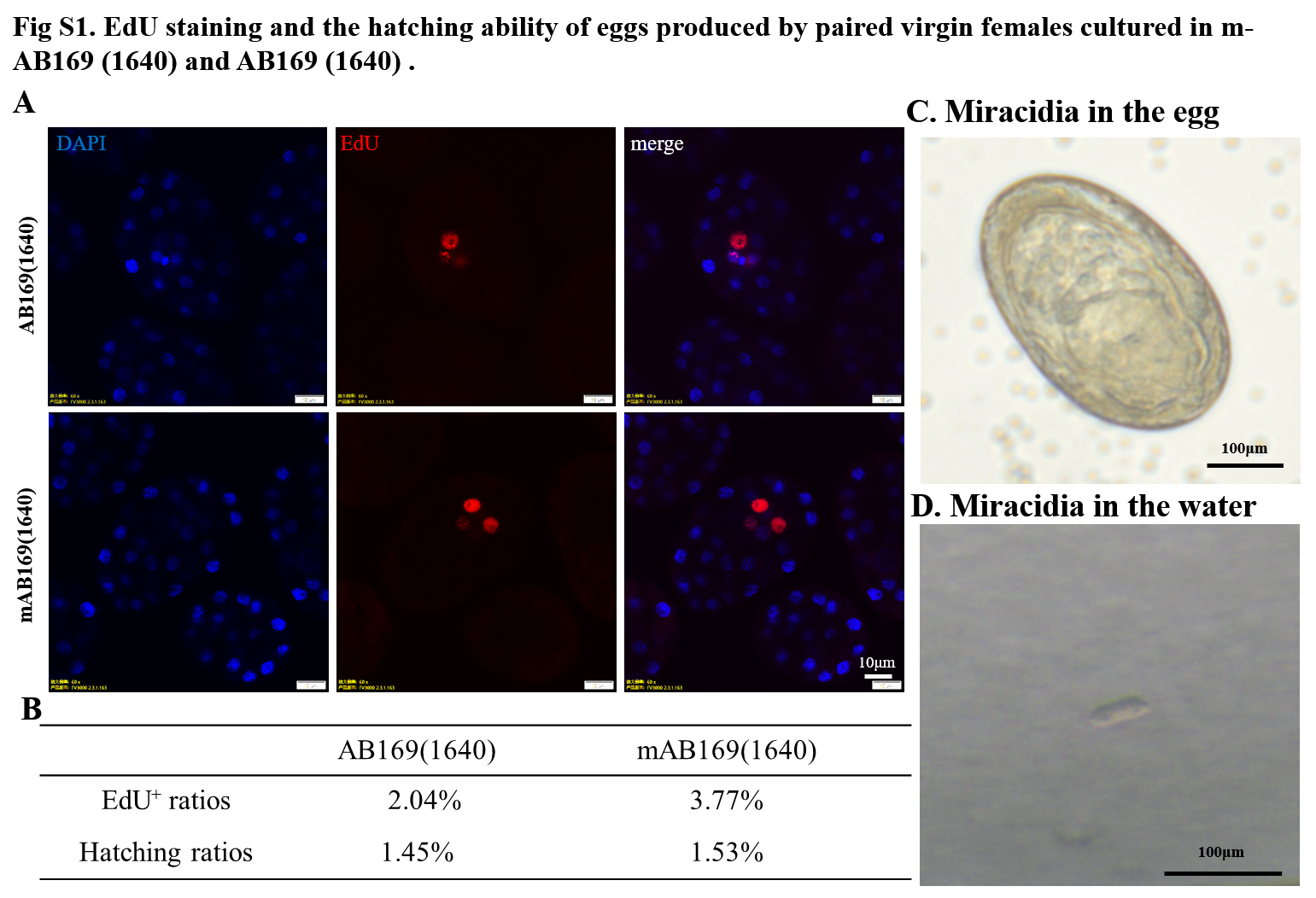

Supplement: Supplementary file 1 — Additional file 1: Figure S1. EdU staining and the hatching ability of eggs produced by paired virgin females cultured in m-AB169 (1640) and AB169 (1640). A EdU staining; B EdU+ and hatching ratios of the eggs. C Miracidia in the egg. D Miracidia hatched from the eggs. [file 13071_2024_6191_MOESM1_ESM.tif]

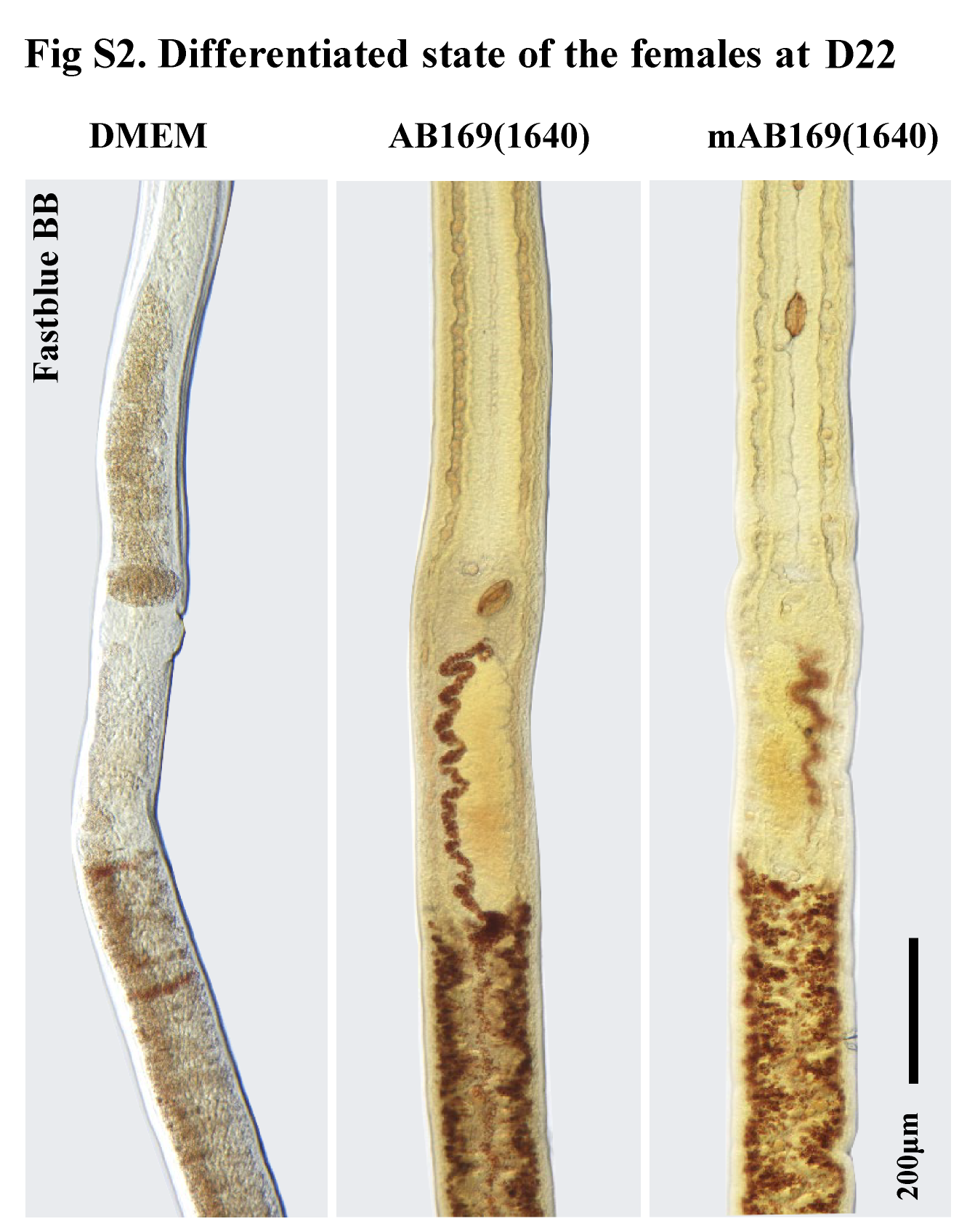

Supplement: Supplementary file 2 — Additional file 2: Figure S2. Differentiated state of the females at D22. [file 13071_2024_6191_MOESM2_ESM.tif]

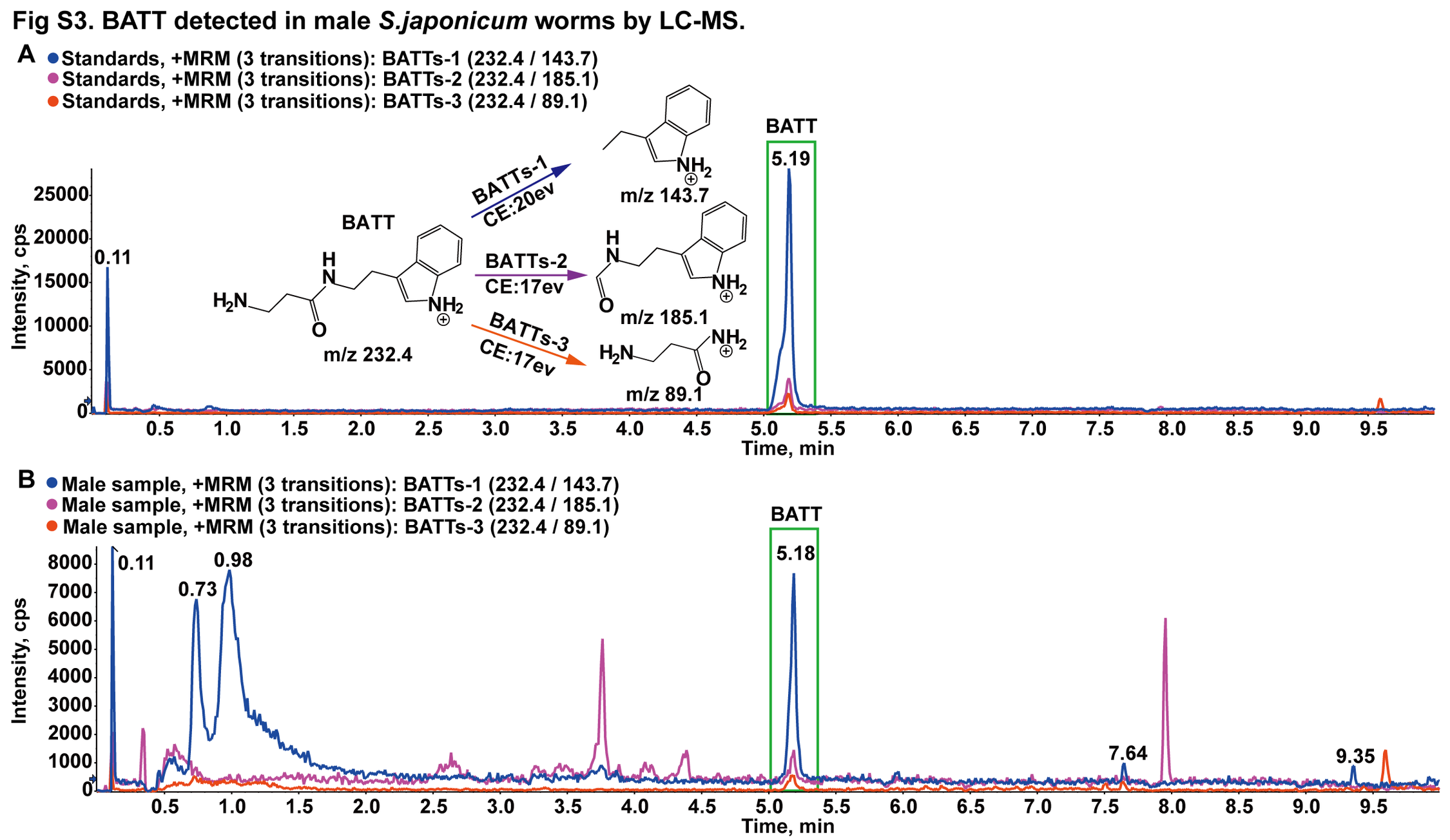

Supplement: Supplementary file 3 — Additional file 3: Figure S3. BATT detected in male S. japonicum worms by LC-MS. A LC–MS detection of m/z at 143.7, 185.1 and 89.1 showing that the retention time (5.19) of the peaks of BATT standards (m/z = 232.4). B LC-MS detection of m/z at 143.7, 185.1, and 89.1 showing that the retention time (5.18) of the peaks from the male S. japonicum extracts. The retention time of the three peaks corresponds to that of the BATT standards. y axis represents intensity, cps, and x axis represents retention time from 0 to 10.0 min. Time is shown for all peaks. m/z, mass-to-charge ratio. Related to Fig. 4. [file 13071_2024_6191_MOESM3_ESM.tif]

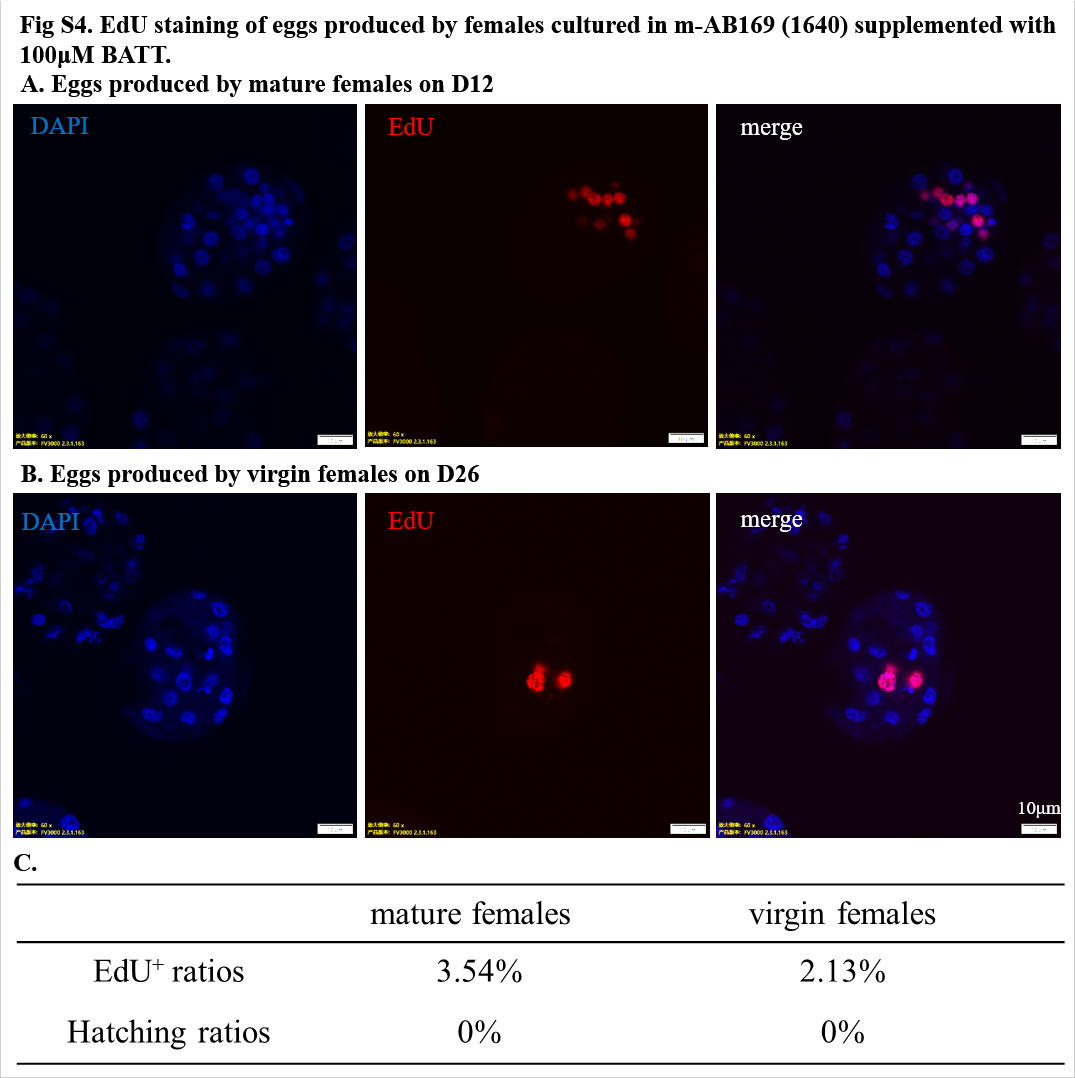

Supplement: Supplementary file 4 — Additional file 4: Figure S4. EdU staining and the hatching ability of eggs produced by females cultured in m-AB169 (1640) supplemented with 100 μM BATT. A EdU staining of eggs produced by mature females on D12; B EdU staining of eggs produced by virgin females on D26. C EdU+ and hatching ratios of the eggs. [file 13071_2024_6191_MOESM4_ESM.tif]

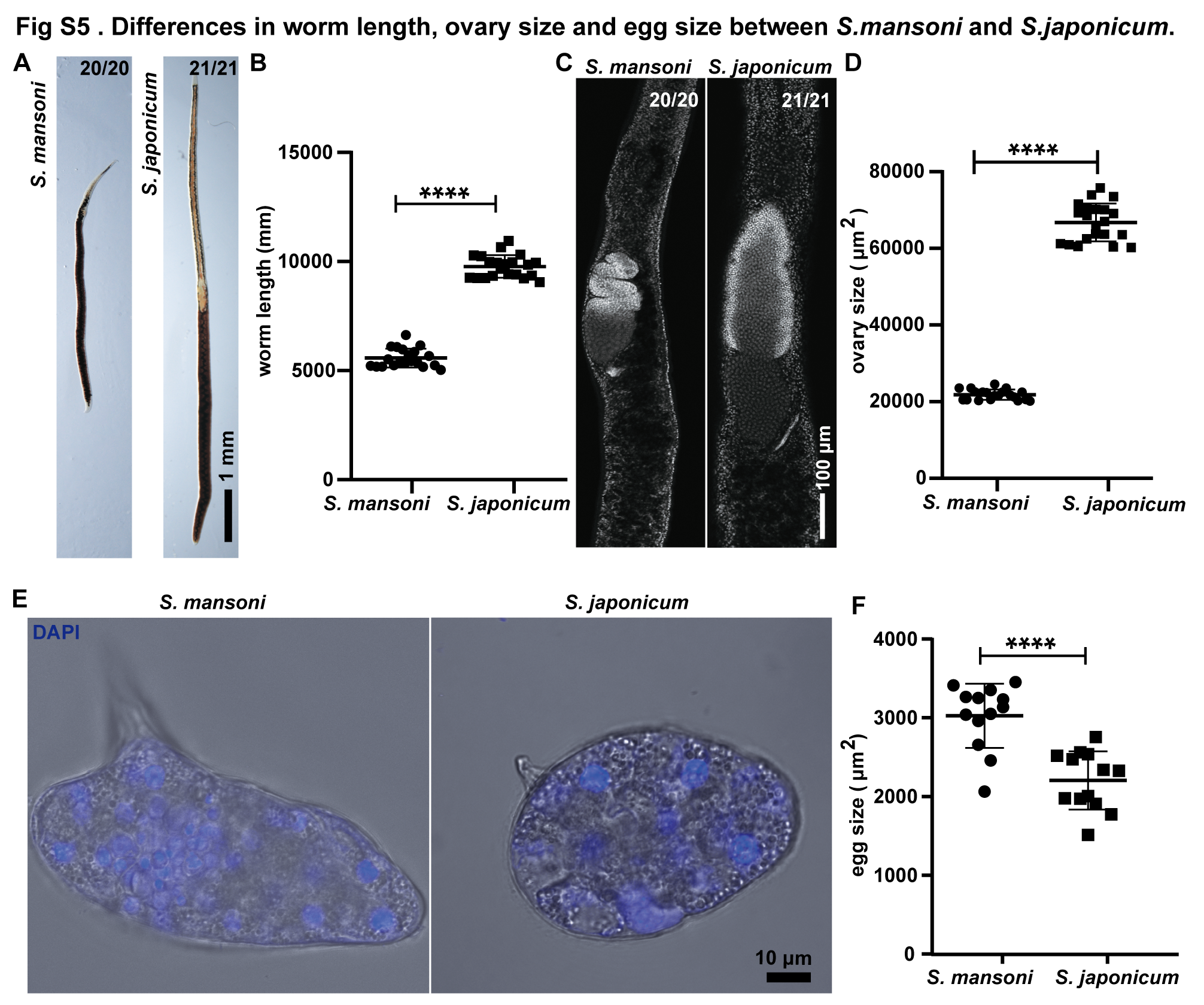

Supplement: Supplementary file 5 — Additional file 5: Figure S5. Differences in worm length, ovary size, and egg size between S. mansoni and S. japonicum. A Brightfield images showing the adult S. mansoni (42 dpi, n = 20) and S. japonicum (34 dpi, n = 21) females stained by Fast Blue BB. B Length of adult S. japonicum (34 dpi, n = 21) and S. mansoni (42 dpi, n = 20) females. C DAPI labeling (gray) showing the ovaries of the adult S. mansoni (42 dpi, n = 20) and S. japonicum (34 dpi, n = 21) females. D The sizes (area) of ovaries in adult S. japonicum and S. mansoni. n ≥ 20 females for each group. E Freshly laid eggs (DAPI-labeled, blue) by adult S. japonicum (34 dpi, n = 21) and S. mansoni (42 dpi, n = 20) on D1 after harvested from the mice. F The sizes (area) of eggs laid by adult S. japonicum and S. mansoni. n = 13 eggs for each group. ****P < 0.0001, t test. Error bars represent the SD. [file 13071_2024_6191_MOESM5_ESM.tif]

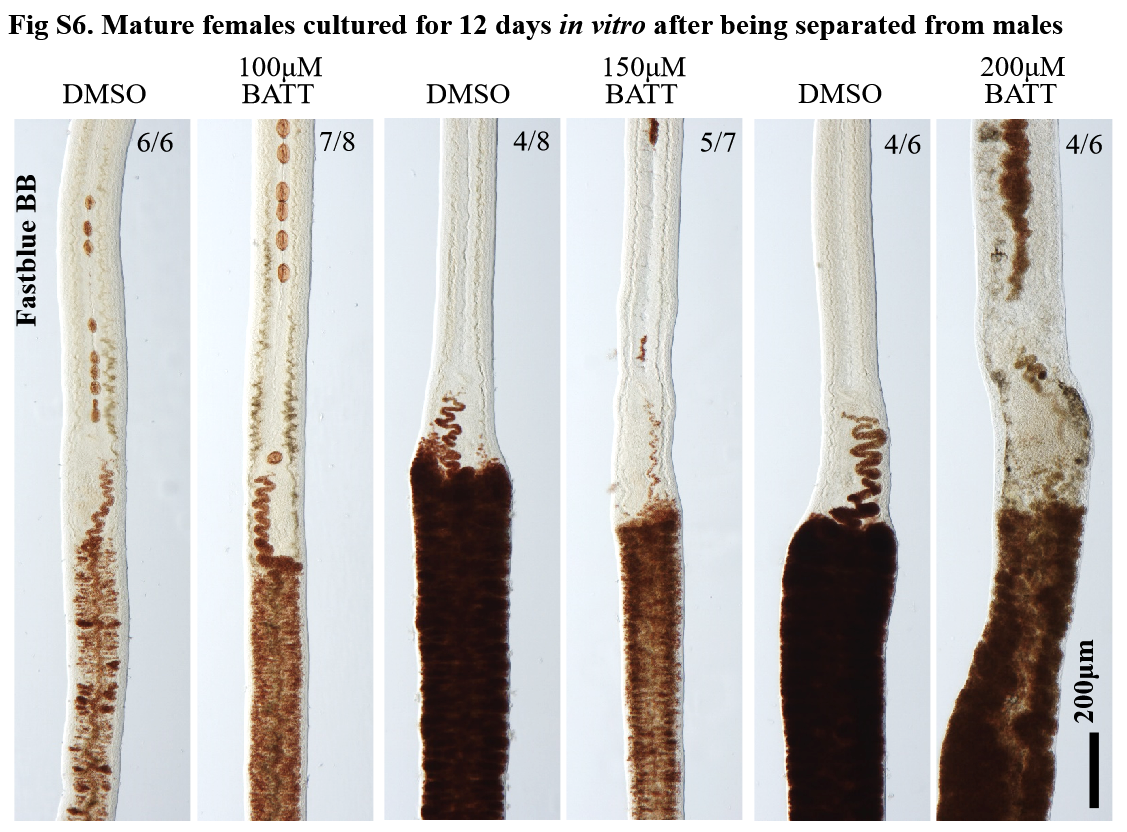

Supplement: Supplementary file 6 — Additional file 6: Figure S6. Mature females cultured (within BATT or DMSO) for 12 days in vitro after separated with males. [file 13071_2024_6191_MOESM6_ESM.tif]
